# Green approach for improving functionality of medical protective textiles using ZnO NPs

**DOI:** 10.1038/s41598-026-39660-8

**Published:** 2026-03-07

**Authors:** Tariq M. Zagloul, Talaat M. Hassan, Naser Gad Al-Balakocy

**Affiliations:** 1 Readymade Garments Department, Faculty of Applied Arts, Damietta University, Damietta, Egypt; 2 Industrial Education Department, Faculty of Education, Helwan University, Cairo, Egypt; 3 Protein & Manmade Fibers Department, National Research Centre, Dokki, Cairo, 12622 Egypt

**Keywords:** Medical textiles, Protective clothing, Cellulase enzyme, ZnO NPs, Antimicrobial, Ultraviolet protection, Biochemistry, Biotechnology, Chemistry, Materials science, Microbiology, Nanoscience and technology

## Abstract

The article explores the possibility of applying enzymatic treatment as a green approach for fabric surface activation that can facilitate loading polyester containing cotton fabrics zinc oxide nanoparticles (ZnO NPs) prepared by sol–gel method. Cotton and PET/C fabrics treated by neutral (Cellu GN 50) and acid (Producto EAPS 55), cellulase enzymes before and after loading with ZnO NPs were investigated by the use of Scanning Electron Microscopy (SEM), Electron Dispersion Emission X-Ray (EDX) and Fourier Transformed Infrared Spectroscopy (FT-IR).The functionality of activated fabrics loaded by ZnO NPs was evaluated by analyzing their antimicrobial activity and UV protection efficacy. Antimicrobial activity of activated fabrics and loaded by sol ZnO NPs was tested against Gram-positive (*Bacillus mycoides*), Gram-negative (*Escherichia coli*), and nonfilamentous fungus (*Candida albicans*). The level of UV protection was verified by the UV Protection factor (UPF) of fabrics. Activated and loaded fabrics with sol ZnO NPs showed outstanding antimicrobial and UV protection efficiency. The achieved antimicrobial function and UV protection on the fabrics are durable with repeated laundering processes even after five washing cycles.

## Introduction

Medical protective clothing for health care workers, specifically to mitigate the risks from exposure to hazardous substances and pathogens, and hence the risk of cross-infections^1^. Researchers provide clothing of health care staff, protection from disease transmission as well as providing comfort and flexibility for ease of work^2^. There has become an urgent necessity for improving fabrics antimicrobial properties to maintain health and reduce infection for suiting medical sector^3^. Medical protective clothing should exhibit necessary characteristics such as antimicrobial properties^4^. However, release of antibacterial agent during use of these fabrics remains a major concern compromising durability of the antibacterial effect and safety of patients and environment^5^.

Polyester containing cotton fabrics are widely used in working textile markets. Because there are a lot of advantages, including pressure resistance, wrinkle resistance, stiffening, moisture absorption, and low cost^6^. However, these fabrics also have some disadvantages, such as tendency to pilling, which affect the appearance and handle of fabrics. It is realized that the problem of pilling is one of the biggest quality issues for polyester/cotton (PET/C) blended fabrics^7^. Garments finishing is the stage of garments production concerned with preparing products for end use, and it refers to a number of operations that have an impact on the quality of the fiber. There are various types of finishing to develop garment finishing and use emerging technologies^8^. The stage of finishing treatment with cellulase is repeating bio-polishing and enzymatic washing processes, which is widely applied in the production of cotton fabrics and denim goods^9^. A major reason for the wide use of enzyme washing is that it is more environmentally friendly than other methods. In the processing of enzyme washing, many parameters such as temperature, duration, and concentration of cellulase enzyme simultaneously affect the results of the process^10^. Recently, cellulase enzyme attract the attention in textile industry due to its role in removing fuzz fibers of cotton fabrics. Cellulase is used in knitted fabric to enhance softness^11^.

Recently, functional finishing of textiles with nanoparticles (NPs) in order to impart them a new functional performance such as antimicrobial^12^, ultraviolet protection^13^ and self-cleaning^14^ for the convenience of consumer and to expand the areas of their use. It is well known that the main factors in attachment NPs with textiles are presence of functional groups such as OH and COOH. Literature was shown that the introduction of additional carboxylic groups to fabrics has likely induced more efficient binding of NPs and more uniform coating of the fiber surface, which in turn resulted in enhanced self-cleaning efficiency. Meilert et al.^15^ have used commercially available nontoxic and low cost saturated polycarboxylic acids as chemical spacers to attach NPs to cotton. Dauod et al.^16^ reported that acylation of wool fibers with non-toxic succinic acid anhydride led to an increase in reactivity toward NPs.

Several recent studies reported the promising potentials of nontoxic and inexpensive ZnO NPs for imparting multifunctional properties to different textile materials^17,18^. The compatibility of TiO_2_ NPs with fiber surface chemical functionalities is one of the most important prerequisites for obtaining stable composite system and long-term durability effects^19^. The tailoring of desirable fiber surface from the standpoint of its chemical functionality and improvement of TiO_2_ NPs binding efficiency has recently gained much scientific interest. Recent studies indicated that treatment of hydrophobic fibers by low-pressure plasma^20^ and corona at atmospheric pressure^21^ can significantly enhance the binding efficiency of ZnO NPs. However, uptill now this approach has not been applied on industrial scale.

Stemming from the above mentioned and from the fact that, industrial wet processing line for natural and man-made fibers includes scouring the fabrics in presence cellulase enzymes, which leads to the creation of additional hydroxyl and carboxyl groups in cotton and PET/C macromolecules, it seems of a great interest to clarify the possibility of applying the enzymatic activation method as a practical alternative to the coating by polycarboxylic acids and plasma approach. Therefore the present work discuses the effect of applying neutral and acid cellulase enzymes, as treatment for fiber surface activation of cotton and PET/C blended fabrics, on its functional finishing with sol ZnO NPs. This suggested finishing technique is expected to paving the way for making them more cost-effective for protective clothing.

## Experimental work

### Materials

*Cotton (100%) and PET/C (50/50) fabrics* used throughout this study were in the form of filament woven fabric cloth made from filament yarns. They were kindly supplied by Misr polyester Co., Kafr El-Dwar, Egypt. The fabrics were scoured at 80 °C for 45 min. with solution containing 2.0 g/l nonionic detergent, washed with cooled water, squeezed, and finally air dried.

*All chemicals* used in this work (Zinc acetate dihydrate), methyl alcohol and sodium hydroxide were purchased from Sigma-Aldrich and have been used as received.

*Cellulases*: Acid enzyme (Producto EAPS 55), Neutral enzyme (Cellu GN 50), Detergent (Asumin wash), from Glorychem, Egypt, the agent of Asutex, Spain.

*Washing machine*: Brand name: Yilmak, Capacity: 5 kg; RPM (Revolution per minute), 30–33 rpm; Origin: Turkey.

*Microorganisms*: *Bacillus mycoides* (Gram positive bacterium), *Escherichia coli* (Gram negative bacterium), and *Candida albicans* (nonfilamentous fungus) were used for estimation of antimicrobial potency of control and treated samples. Microorganisms were obtained from the culture collection of the Department of Microbial Chemistry, Division of Genetic Engineering and Biotechnology, National Research Centre of Egypt.

*Culture medium*: Modified nutrient agar medium was used and is composed of the following ingredients (g/L): peptone (10.0), beef extract (5.0), NaCl (5.0), and agar (20.0). The pH was adjusted to 6.8. This medium was sterilized for 20 min., at 121 °C under pressure.

### Method

*Rinse*: All of fabric samples were treated with detergent (Asumin wash) in the following bath: detergent 1.0 g/l at pH 7, room temperature, time 5 min., and material to liquor ratio, 1: 10.

*Activation by acid cellulase*: The fabric samples were treated with acid cellulase enzyme (Produto EAPS 55) in the following bath: acid cellulase enzyme 1.0%, and acetic acid 1.0 g/l at pH 4.5, temperature 50 °C, duration for 30 min., and material to liquor ratio 1: 10. After the treatment was finished, the samples were raised up to 70–80 °C for 10 min., to deactivate the enzyme activity. Then samples were rinsed by cold water in a bath for 10 min.

*Activation by neutral cellulase*: The fabric samples were treated with neutral cellulase enzyme (Cellu GN 50) in the following bath: neutral cellulase enzyme 1.0% at pH 7, temperature 50 °C, time 30 min., and material to liquor ratio 1: 10. After the treatment was finished, the samples were raised up to 70–80 °C for 10 min. to deactivate the enzyme activity. Then samples were rinsed by cold water in a bath for 10 min.

*Preparation and loading fabrics by Sol ZnO NPs*: The loading of activated cotton and PET/C blended fabrics with the cellulases was carried out using a high-temperature LL—high-pressure laboratory dyeing machine -India. Activated cotton and PET/C blended fabrics used in the suggested method as a medium for synthesis ZnO NPs. the fabric samples were immersed in 0.09 molar zinc acetate aqueous solution (Material—to—liquor ratio (M:L); 1:50 at 50 °C for 30 min. Solution temperature was increased by temperature gradient of 2 °C/min up to 95 °C and followed for 30 min. After that, they were treated with NaOH aqueous solution at twice molar concentration at 50 °C for 30 min and followed at 95 °C for 30 min., then rinsed with distilled H_2_O. finally, the finished fabric samples were dried at 100 °C for 30 min. and cured at 130 °C for 5 min.^22^. In order to evaluate the durability (binding ability) of sol ZnO NPs to the fabrics, the finished samples were washed five washing cycles according to the standard method AATCC test method (61-1989).

### Analysis

#### Antimicrobial activity

Antimicrobial activity of PET and PET/C blend fabrics loaded with ZnO NPS was quantified using the following method. Shake Flask method: in this case the antimicrobial activity of immobilized antimicrobial agents is determined under dynamic contact conditions according to ASTM standard test method 2149 (2001)^23^.

### SEM and EDX

Surface structure and the morphology of all fabric samples characterized by a JEOL-Model JSM T20 scanning electron microscope (SEM), operating at 19 kV was used to obtain photomicrographs of fabrics surfaces.

### FT-IR

FTIR analysis has been performed utilizing Fourier transform infrared spectrometer model V80 from Brukeroptics, Deutschland. The total internal reflection technique has been employed with spectral resolution 2 cm^−1^. Every spectrum has been recorded 5 times and averaged, smoothed, and baseline-corrected using a self-written subroutine.

Raman analysis has been performed utilizing a con focal Raman microscope from Witech-Oxford, Deutschland. The excitation wavelength was 532 nm from a picoseconds pulsed laser source. Raman spectra have been collected using 60X UP lanZeiss objective, for 120 s. with laser power 50 mW.

### UPF factor

UPF factor was measured using UV- Shimadzu 3101 P C -Spectrophotometer. It is a double beam direct ratio measuring system. It consists of the photometer unit and a pc computer. UPF factor was determined according to the method described in Australian/ New Zealand standard AS / NZS 4399: 1996^24^.

## Results and discussion

### Effect of cellulase enzymes on cotton component

Commercial cellulases for bio-polishing originate from Trichodermareesei and Humicola insolens^25,26^. Acid cellulases are Trichoderma based products which work best at pH 4.5–5.5 at a temperature of 45–55 °C^27^. Neutral cellulases from Humicola are more effective at pH 6.0–7.0 and in the temperature range of 40–55 °C^28,29^. The use of acid cellulases, in bio-stoning is limited due to the back staining and weakening of fabrics they cause^30^. Neutral cellulases, with activities in the pH range 6–7, are less aggressive against cotton than acid cellulases, and do not compromise the strength of the fabric as readily as acid cellulases^31^. However, neutral cellulases generally require a longer wash time than do the acid cellulases^30^. Abrasion resistance was improved due to bio-polishing in acid cellulase treatment showed better resistance than the neutral treatment^32^.

It is clear from tables (1 and 2) that, cotton and PET/C fabrics treated with neutral cellulase had the lowest hydrolysis rate with 2.8 and 2.3 weight loss %, while enzymatic treatment with acid cellulase brings about a noticeable decrease in loss the weight of cotton and PET/C blend fabrics, 4.2 and 3.5 weight loss % respectively^33^. Cellulases has a higher specific activity towards cotton component than ester molecules; this explains the higher weight loss with cotton than PET/C fabric, a higher specific activity correlates with higher weight loss % with cotton fabric. This is a direct consequence of a partial enzymatic hydrolysis of the cellulosic fibers especially on the fabric surface and amorphous regions, yielding soluble products such as short-chain oligomers and glucose^34^.

### Formation and loading of sol ZnO NPs on activated fabrics

ZnO NPs were generated insitu on the surface of cotton and PET/C fibers to serve as a soft template in the synthesis process. Cotton and PET/C fibers contain negative charges in neutral and alkaline aqueous solutions due to the ionization of COOH end groups throughout the polymer chain of PET molecules and the presence of OH groups on the methylol side chains of glucose molecules of cotton^35^. Negative charges on the adsorbing surface of treated fibers are thought to be attractive sites for cation absorption. As a result of electrostatic interactions, these groups may formed ionic bond with Zn^2+^ metal ions from the padding solution. When the fabric samples were immersed in an alkaline solution, the absorbed zinc ions were transformed into zinc atoms, which grew to form nanoparticles^36^. The absorption mechanisms of zinc ions onto fibers and insitu ZnO NPs generation are discussed using the following equations.

1 $$\left[ {{\mathrm{PET}}/{\text{C or Cotton}}} \right]{-\!\!-}{\mathrm{XH}}^{ - } \to {\mathrm{Cotton}}\mathrm{-}{\mathrm{X}}^{ - } , ({\mathrm{XH}} = {\text{OHand COOH}})$$

2 $$\left( {{\mathrm{CH}}_{{3}} {\mathrm{COO}}} \right)_{{2}} {\mathrm{Zn}} \to {\mathrm{2CH}}_{{3}} {\mathrm{COO}}^{ - } + {\mathrm{Zn}}^{{2+}}$$

3 $${\mathrm{PET}}/{\text{C or Cotton}}{-\!\!-}{\mathrm{X}}^{ - } + {\mathrm{Zn}}^{{2+}} \to {\mathrm{Cotton}}{-\!\!-}({\mathrm{X}}^{ - } )_{{2}} {\mathrm{Zn}}^{{2+}}$$

4 $${\mathrm{NaOH}} \to {\mathrm{Na}}^{ + } + {\mathrm{OH}}^{ - }$$

5 $${\mathrm{Zn}}^{{2+}} + {\mathrm{2OH}}^{ - } \to {\mathrm{Zn}}({\mathrm{OH}})_{{2}}$$

6 $${\mathrm{Zn}}({\mathrm{OH}})_{{2}} \to {\mathrm{ZnO}} + {\mathrm{H}}_{{2}} {\mathrm{O}}$$

**Scheme 1:**
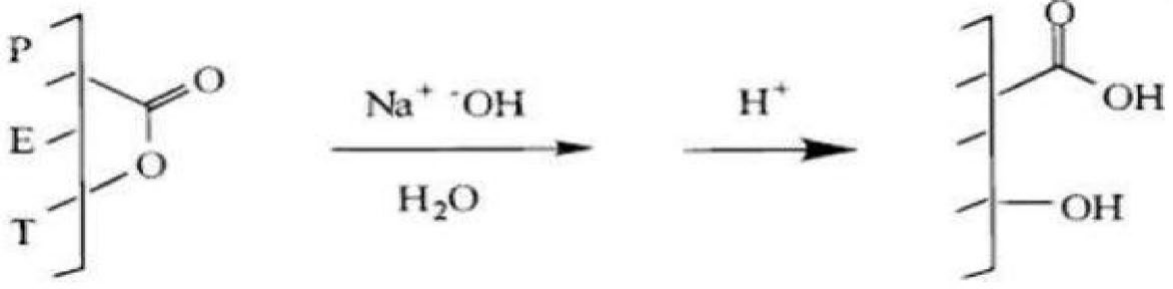
Effect of Alkali on Polyethylene terephthalate (PET)^37^.

Insitu synthesis of ZnO NPs in aqueous alkaline medium and finishing cotton and PET/C blended fabrics has been used to enhancement the attachment of NPs to the surface of fabrics. This treatment leads to a partial hydrolysis of the surface fabric and creates new groups on PET macromolecules (Scheme 1). This may be resulting from, alkaline treatment causes partial hydrolysis of some ester groups which accompanied by a significant increase in OH and COOH groups on the surface of polyester fibers. These situation were experimentally proved by the estimation of functional groups creating on the surfaces of PET/C blended fabrics before and after the finishing with sol ZnO NPs through carboxylic content measurement (Table 2).

**Fig. 1:**
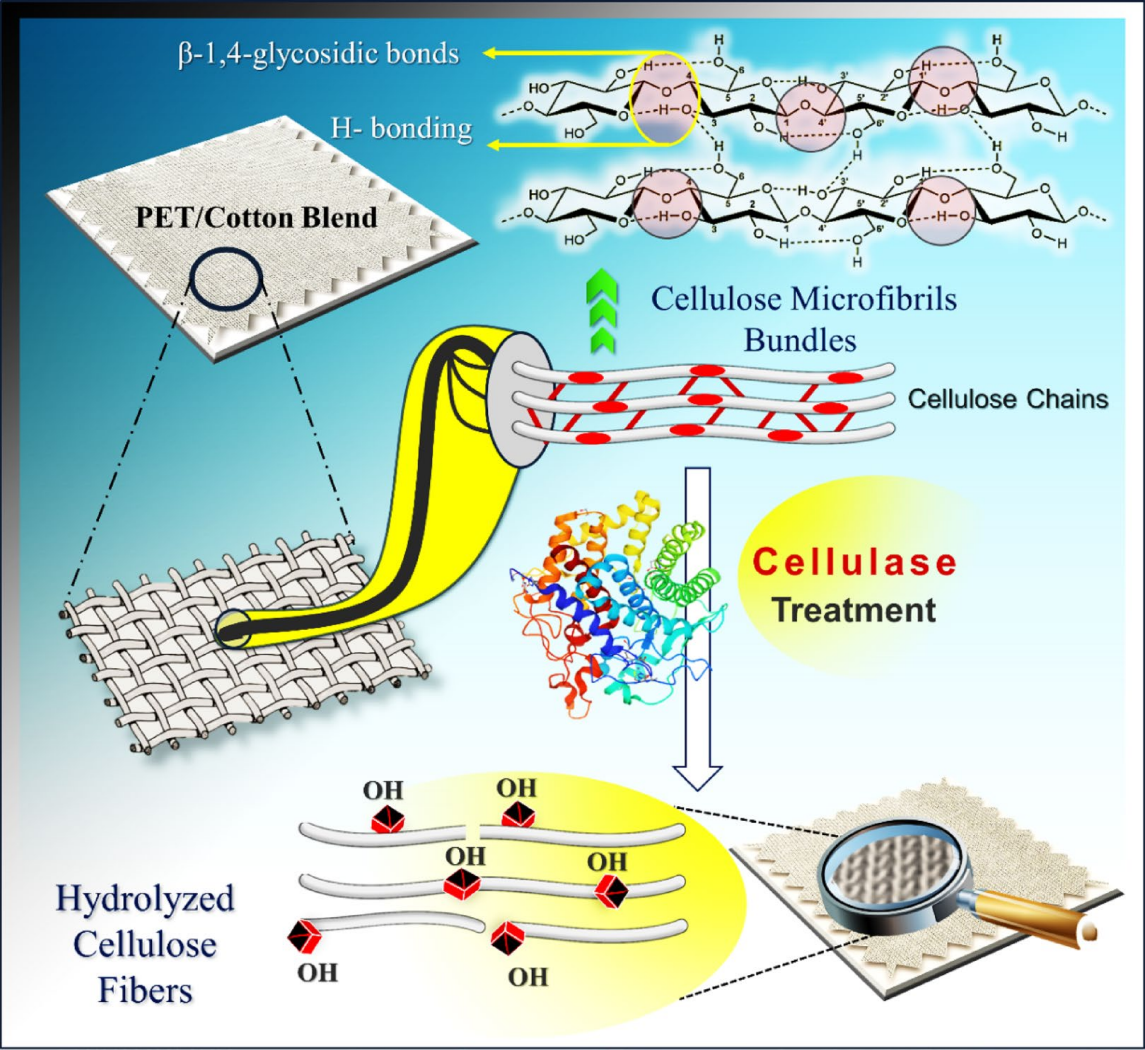
Effect of cellulases on cotton fibers.

The cellulases treatment and insitu sol finishing is accompanied by an increase in the carboxylic and hydroxyl content of cotton and PET/C blended fabrics (Fig. 1) resulting from partial hydrolysis of glycosidic bonds. Therefore, the extent of sol ZnO NPs increase by increasing the amount of carboxylate groups. The atomic weight % of ZnO value is higher, the greater the loss in weight, regardless of the used fabrics.

### Characterization of activated fabrics loaded with ZnO NPs

#### Surface topography (SEM)

**Fig. 2:**
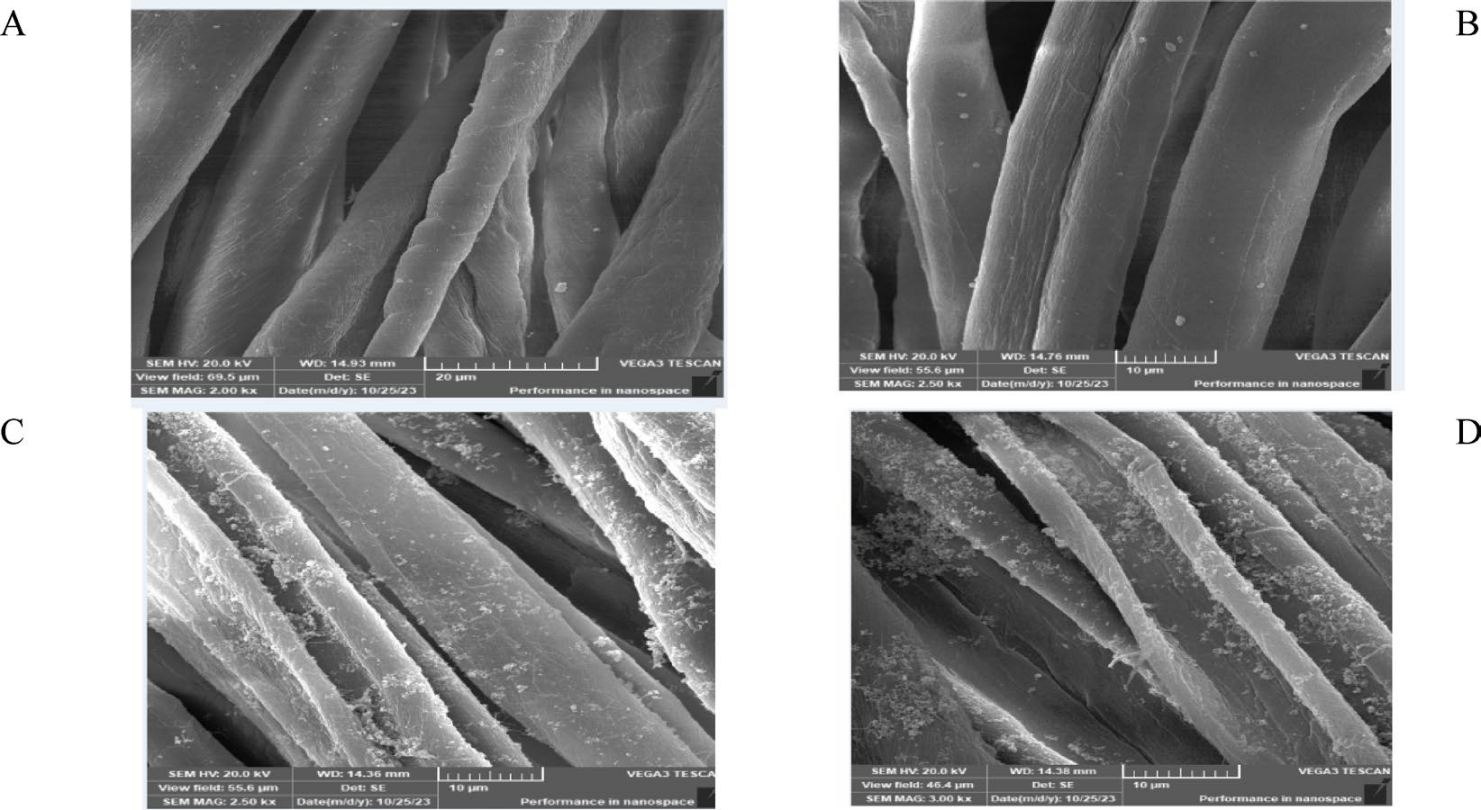
SEM Micrographs of Activated cotton Fabrics* and Loaded with sol ZnO NPs (1000x). (**A**) Cotton → Acid Cellulase; (**B**) Cotton → Neutral Cellulase; (**C**) Cotton → Acid Cellulase → ZnO NPs; (**D**) Cotton → Neutral Cellulase → ZnO NPS. *After Five Washing Cycles According to AATCC Test Method (61-1989).

**Fig. 3:**
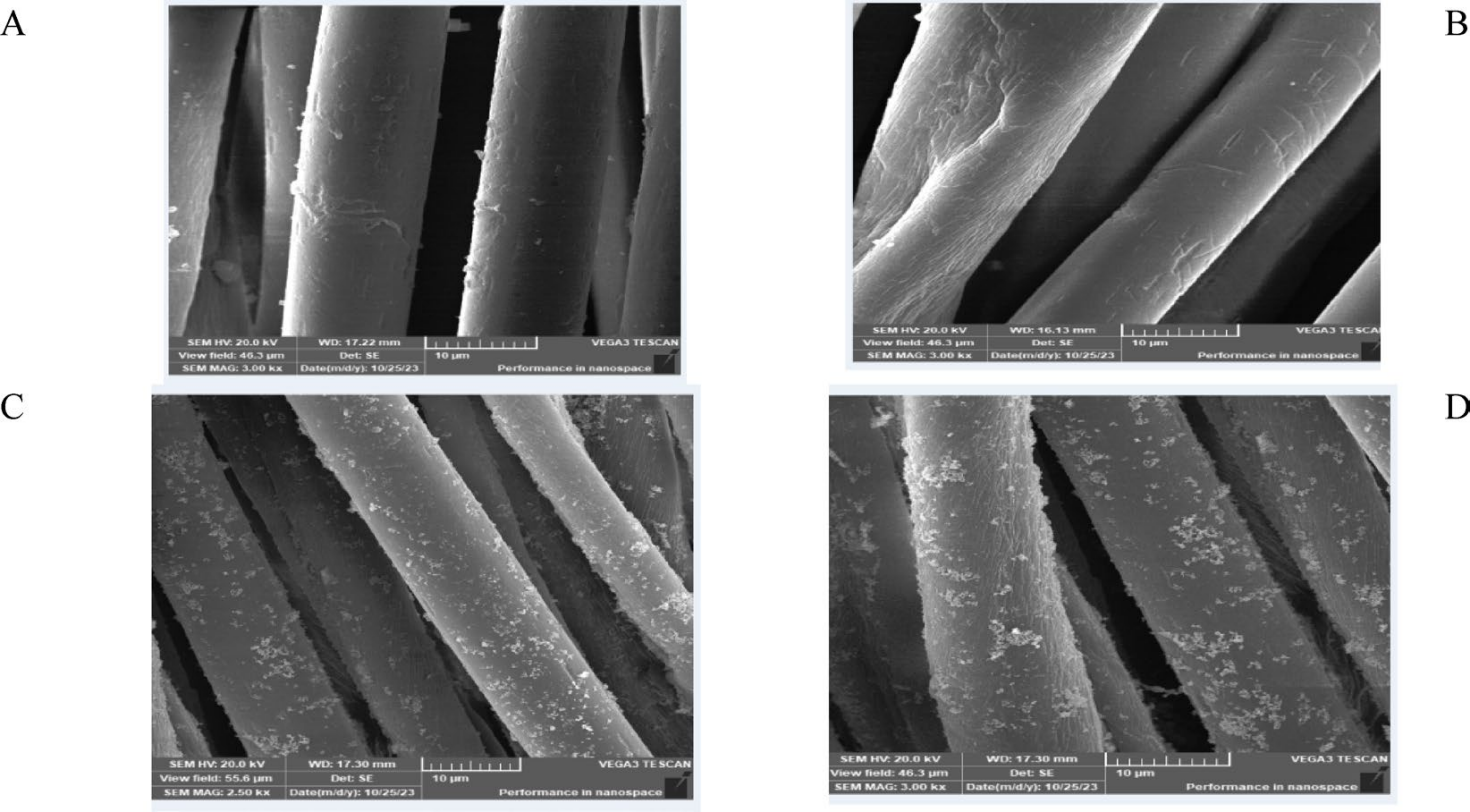
SEM Micrographs of Activated PET/C blended Fabrics* and Loaded with sol ZnO NPs (1000x). (**A**) PET/C → Acid Cellulase; (**B**) PET/C → Neutral Cellulase ; (**C**) PET/C → Acid Cellulase → ZnO NPs; (**D**) PET/C → Neutral Cellulase → ZnO NPS. *After Five Washing Cycles According to AATCC Test Method (61-1989).

In order to investigate the morphology of the activated fabrics with cellulases and loaded by ZnO NPs, SEM images of samples were recorded in Figs. 2 and 3. The images have been done anto cotton and PET/C fabrics activated and loaded with sol ZnO NPs followed by five washing cycles. Figures 2A, B and 3A, B showed that the surfaces of cotton and PET/C blended fabrics treated by cellulases are clean and smooth. This is due to, the amount of weight reduction along with elimination of hairiness on the fabric surface thereby minimizing stiffness and thickness as well as imparting a smooth surface.

The treated fabrics by cellulases and loaded with ZnO NPs by sol–gel Figs. 2C, D and 3C, D are covered by a thinner uniform surface layer; a continuous deposited material is shown clearly. PET/C blended fabric activated by cellulase and loaded with sol NPs showed a few pits were appeared on the PET fibers surfaces, the latter have gained a roughness fabric surfaces (Fig. 3C, D) due to the effect of finishing fabrics by sol ZnO NPs prepared in aqueous alkaline medium. The treatment with ZnO leads to blocking some of these defects and formation of thin layer of active substrate on the fiber surface. The treatment of the fabrics with ZnO leads to the formation of some deposits on the surface of treated fabrics. The shape and the size of such deposits vary according to the fabrics used during the enzymatic treatment.

#### EDX

**Fig. 4:**
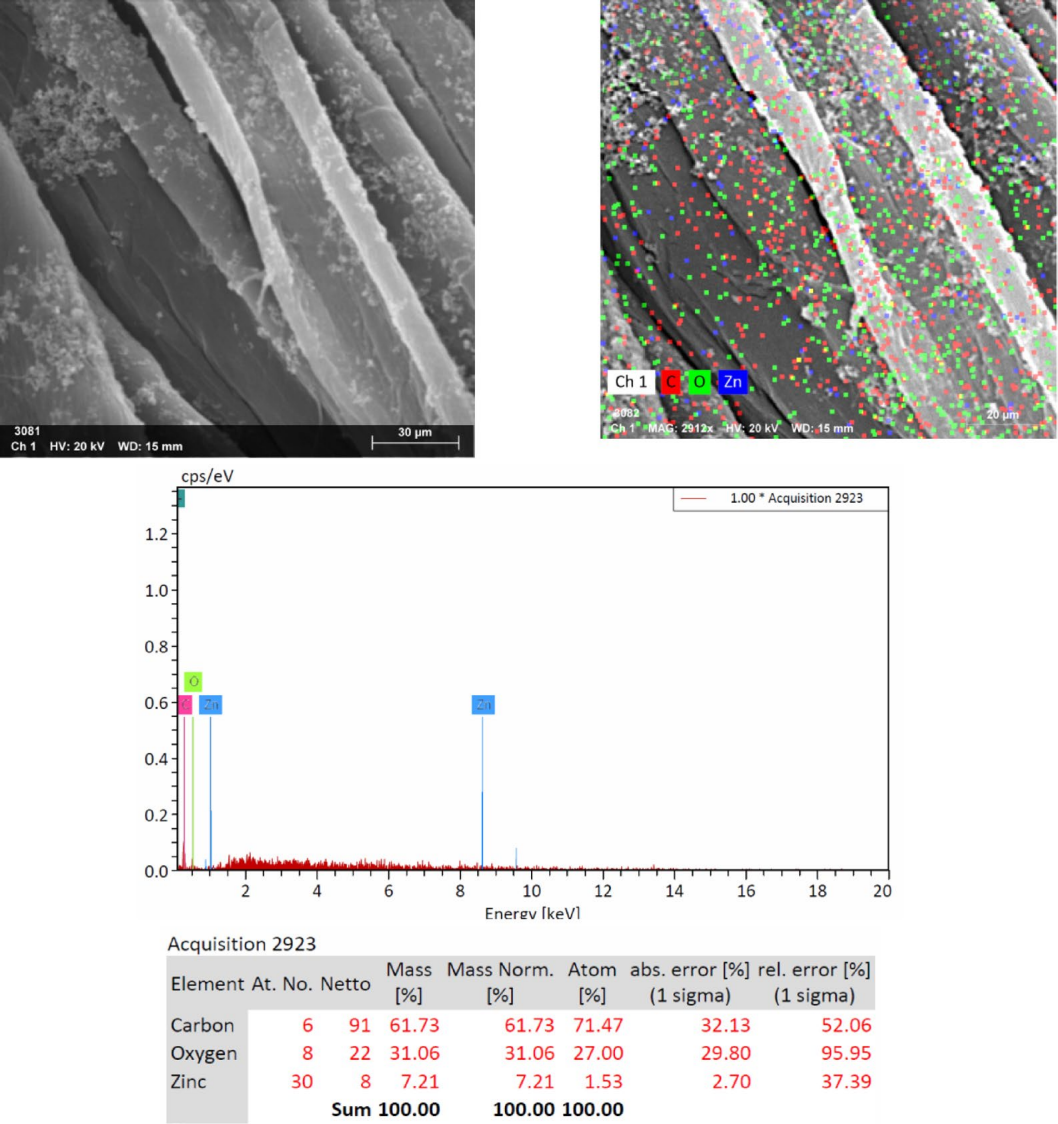
EDX and Mapping of Zinc and Oxygen elements for acid cellulase treated cotton fabric* and loaded with sol ZnO NPs. *After Five Washing Cycles According to AATCC Test Method (61-1989).

**Fig. 5:**
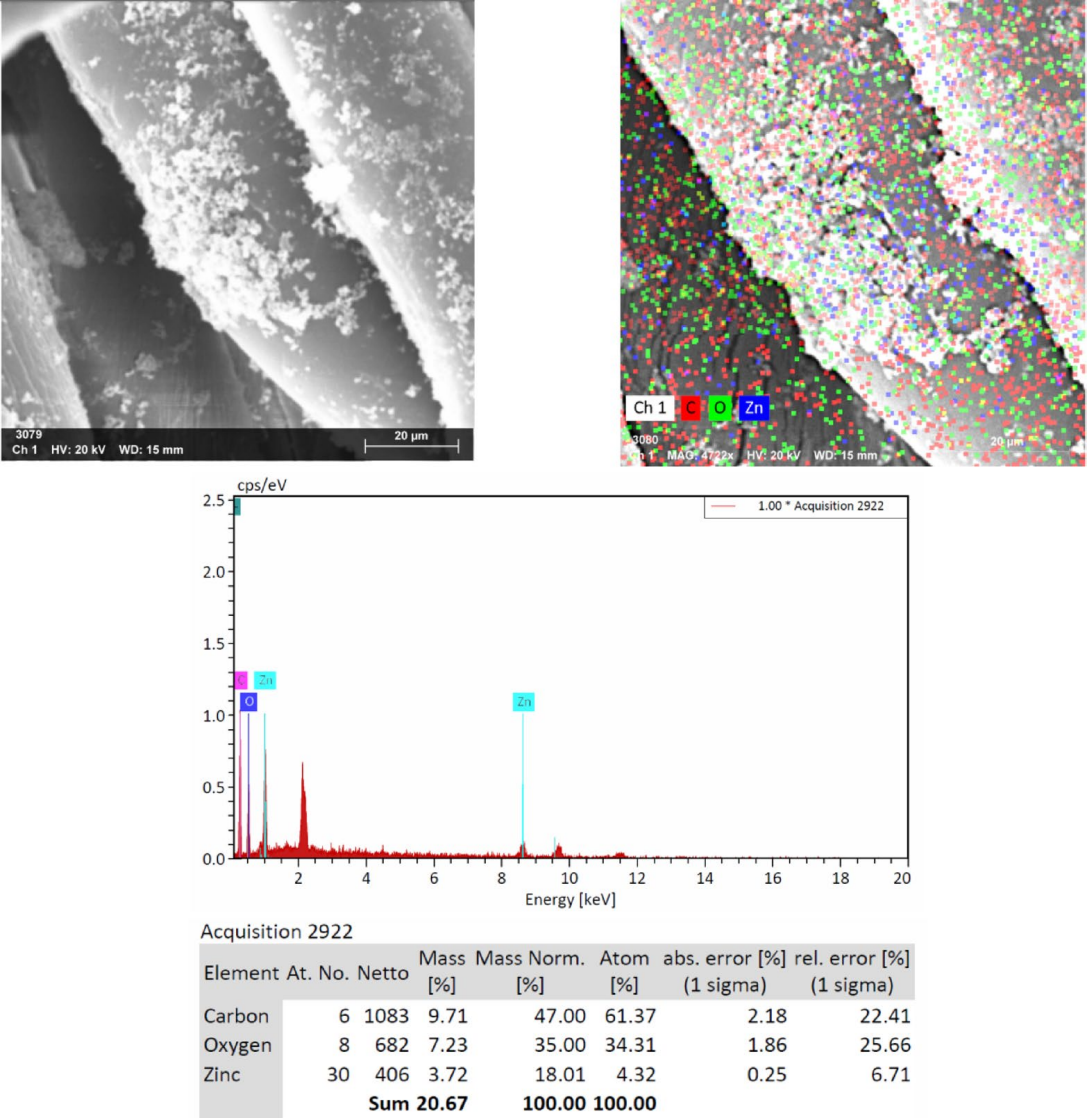
EDX and Mapping of Zinc and Oxygen elements for the cellulase treated PET/C blended fabric* and loaded with sol ZnO NPs. *After Five Washing Cycles According to AATCC Test Method (61-1989).

The presence of ZnO NPs on the surface of fabrics was confirmed by EDX analysis. EDX spectra of the fabrics loaded with ZnO NPs after five washing cycles are shown in Figs. 4 and 5. On the basis of these spectra, it is noteworthy to conclude that the deposited material consisted of Zn and oxygen. This shows that even after five washing cycles (25 home washings), ZnO is still present on the fabrics surface. The surface topography of cotton and PET/C blended fabrics was investigated using EDX technique. Finishing of fabrics activated by acid or neutral cellulases with ZnO NPs is also accompanied by the formation of precipitates (Fig. 1). This is a direct indication on the presence of ZnO NPs on the fiber’s surface (Tables 1 and 2). The above mentioned changes which took place on the surface topography of cotton and PET/C blended fabrics loaded with sol ZnO NPs are confirmed that the ZnO NPs are directly attached to the fabrics surfaces.

**Table 1:** Effect of the cellulases treatment on the amount of weight loss % and ZnO NPs Loaded on Cotton fabrics*

Fabric	Weight Loss %
Cotton (Parent)	0.0
Cotton → N-Cell (Control)	2.8
Cotton → A-Cell (Control)	4.2
Cotton → N-Cell → ZnO NPs	2.8
Cotton → A-Cell → ZnO NPs	4.2

Enzymatic Treatment Conditions: [Acid Cellulase]: 1%, [Acetic Acid], 1.0 g/l, pH = 4.5, Time, 30 min., Temperature, 50  °C, M: L, 1:10. [Neutral Cellulase]: 1%, pH = 7, Time, 30 min., Temperature, 50  °C, M: L, 1:10.

Sol–gel Treatment Conditions: Curing Temperature, 150˚C; Curing Time, 15 min.

* Washed one cycle according to AATCC Test Method (61-1989).

**Table 2:** Effect of the cellulases treatment on the amount of weight loss %, carboxylic content and ZnO NPs Loaded on PET/C blended fabrics*

Fabric	Weight Loss %	Carboxylic Content (meq/100 gr. Fabric)
PET/C (Parent)	0.0	14.3
PET/C → N-Cell (Control)	2.3	18.5
PET/C → A-Cell (Control)	3.5	21.7
PET/C → N-Cell → ZnO NPs	2.3	26.3
PET/C → A-Cell → ZnO NPs	3.5	31.2

Enzymatic Treatment Conditions: [Acid Cellulase]: 1%, [Acetic Acid], 1.0 g/l, pH = 4.5, Time, 30 min., Temperature, 50 °C, M: L, 1:10.

[Neutral Cellulase]: 1%, pH = 7, Time, 30 min., Temperature, 50 °C, M: L, 1:10.

Sol–gel Treatment Conditions: Curing Temperature, 150˚C; Curing Time, 15 min.

* Washed one cycle according to AATCC Test Method (61-1989).

Figures 4 and 5 showed the physical characteristics as well as the chemical composition of the cotton and PET/C blended fabrics activated by acid cellulase and loaded with sol ZnO NPs: Figs. 4 and 5, Zn elemental compositions of the cotton and PET/C fabrics were recorded by EDX. The determined atomic mass % in case of cotton was found 7.21 and 3.72 in case of PET/C blended fabric. The element mapping (Figs. 3, 4) revealed that the Zn NPs spread uniformly within the fabric surface. EDX method used to identify the chemical composition of a specific substance with great precision. EDX measurements also reveal higher Zn content on hydrolyzed and treated cotton fabrics by acid cellulase more than PET/C blended fabric treated by acid cellulase (Zn atomic weight % was 7.21 in case of cotton fabric, on the other hand 3.72 with PET/C fabric). This means that ZnO NPs have sufficient adhesion towards the activated fabrics either by acid or by neutral cellulases and the higher weight reduction was accompanied by greater attached amount of sol ZnO NPs.

#### FT-IR

**Fig. 6:**
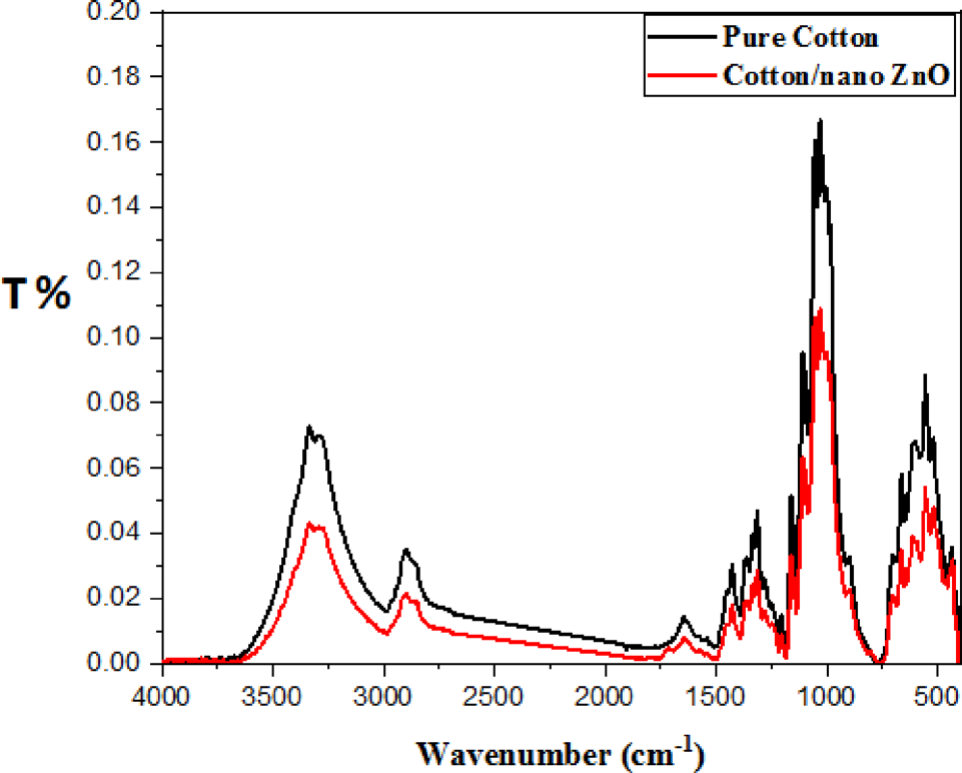
FTIR curves of PET/C blended fabrics* activated by acid cellulase and loaded with sol ZnO NPs. * Washed one cycle according to AATCC Test Method (61-1989).

**Fig. 7:**
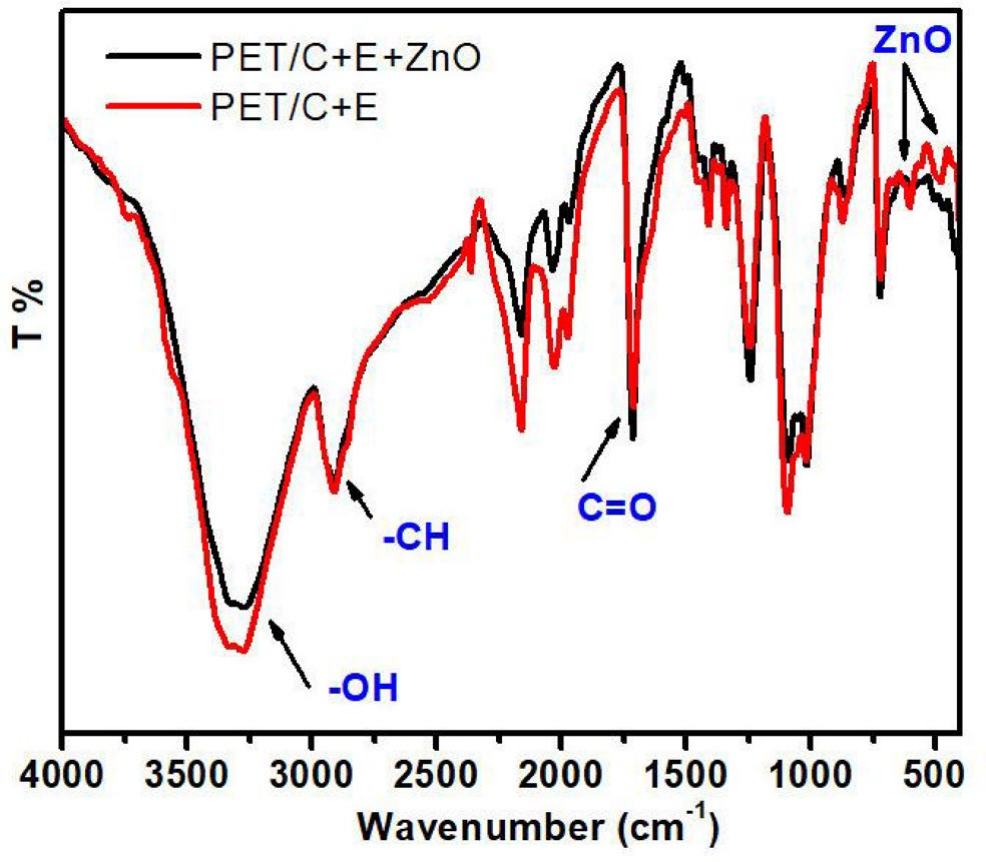
FTIR curves of PET/C blended fabrics* activated by acid cellulase and loaded with sol ZnO NPs * Washed one cycle according to AATCC Test Method (61-1989).

Evidently, acid cellulase treatment before activation induced a significant change in the chemical structure of the fabrics under investigation. The FTIR spectrum (Figs. 6, 7) of parent cotton and PET/C fabrics showed absorption bands at 1649–1712, 3408–3388, and 2317 cm^−1^, which are typical to those of C=O, OH, and CH stretching respectively. New band at 640 was appeared in the pattern of cotton fabric activated with cellulases which can correspond to Zn–O of the new bond cotton—ZnO^38^. The presence of such bands can confirm the ionic interaction of the new bond formed due the addition of sol ZnO NPs to cellulases activated cotton fabric (Fig. 5).

The FT-IR spectrum of activated PET/C blend fabric with acid and/or neutral cellulases and loaded by sol ZnO NPs (Fig. 7) shows that new characteristic peak was showed and located at around 665 cm^−1^. These peak was corresponding to Zn–O bonds of the new bond PET/C—ZnO. The similar finding was reported in^39^. During this study we found that only activated fabrics with cellulases were able to attach with ZnO NPs from sol solutions.

**Scheme 2:**
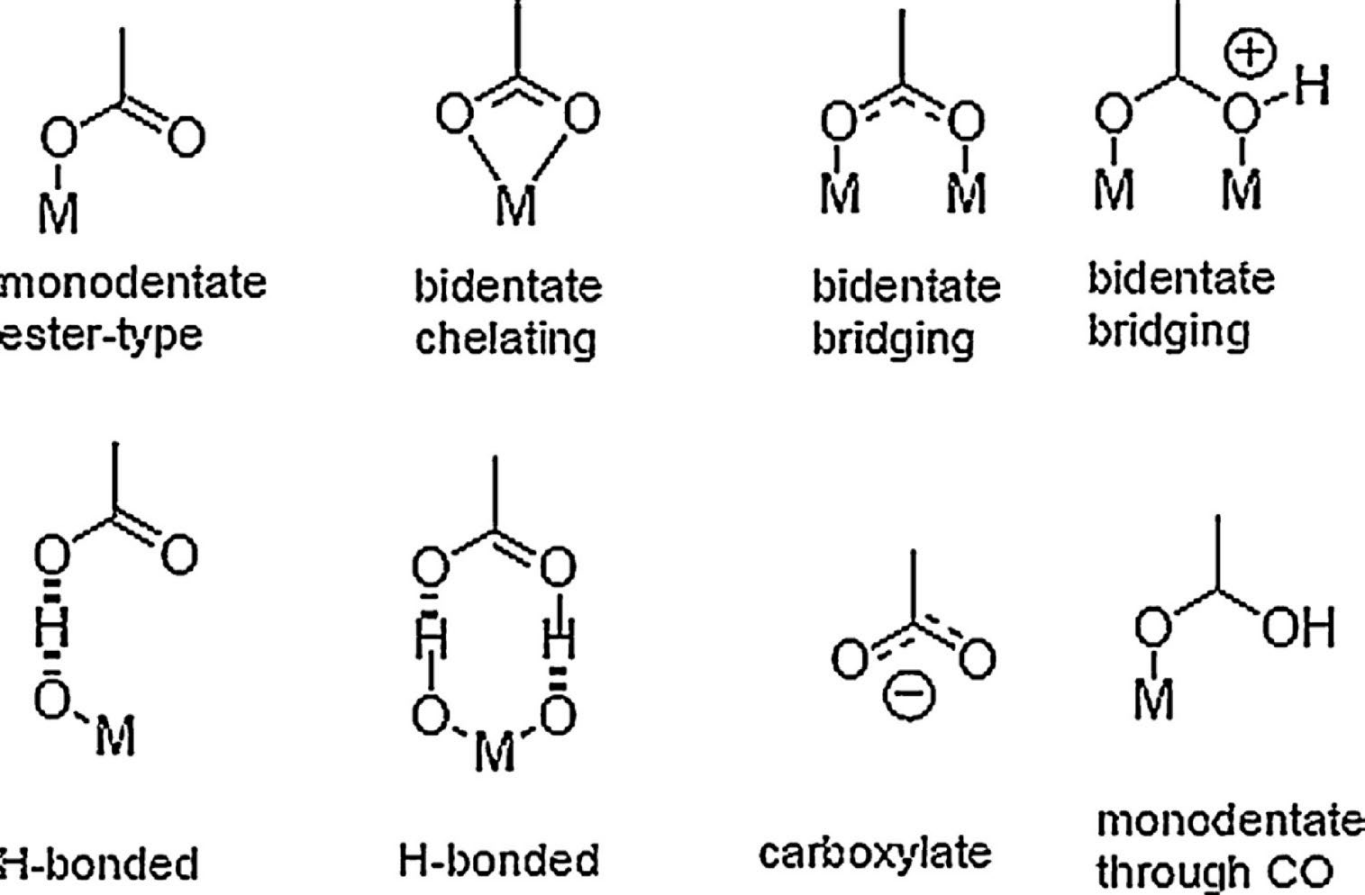
Possible binding mode of a COOH group to NPs.

It is well known that metal (M) atoms can be bound to carboxyl and hydroxyl groups through different modes which are shown in scheme 2^40^. Carboxyl groups can be bound in a mono-dentate mode to form an ester like linkage or they can bind with each of their two oxygen atoms either to one metal atom (bi-dentate chelating) or to two of them (bi-dentate bridging). In addition, they can interact with the metal surface through hydrogen bonding either with a surface bound hydroxyl group and/or a lattice oxygen atom^41^.

### Antimicrobial activity

The antimicrobial activity of cotton and PET/C blend fabrics activated with acid cellulases on one hand, and with neutral cellulases on the other hand, followed by loading with sol ZnO NPs, was investigated against Gram-positive *B. mycoides*, Gram-negative, *E. coli* and non-filamentous fungus *C. albicans* (Tables 3 and 4). The activity was evaluated by determining the reduction percentage of colony forming units (% CFU) of the mentioned microbes using the shake flask method. Table 3 indicates the antimicrobial potency of cotton fabric loaded with ZnO NPs after enzymatic activation. It is clear from the data showed is this Tables 3 and 4 that, all fabrics showed, after 5 washing cycles, high antimicrobial activity against the previously mentioned three microorganisms. In fact, the % CFU for all tested samples are significant, whereas no effect is found for all parent and control samples. The role of activation of fabrics with cellulases before loading with sol Zn ONPs on the antimicrobial activity seems to be more significant as the samples were laundered repeatedly in launder-Ometer. This proves the feasibility of using the cellulases for activation of cotton and PET/C blend fabrics before finishing with sol ZnO NPs.

**Table 3:** Effect of activation of cotton fabrics on its antimicrobial activity.

Reagents	Antimicrobial Assessment		
	*Staphylococcus* *aureus*	*Escherichia* *coli*	*Candida albicans*
Cotton (Parent)	−ve	−ve	−ve
Cotton → N-Cell (Control)	−ve	−ve	−ve
Cotton → A-Cell (Control)	−ve	−ve	−ve
Cotton → N-Cell → ZnO NPs	89.9	82.1	86.7
Cotton → A-Cell → ZnO NPs	91.6	89.5	90.3

Enzymatic Treatment Conditions: [Acid Cellulase]: 1%, [Acetic Acid], 1.0 g/l, pH = 4.5, Time, 30 min., Temperature, 50 °C, M: L, 1:10. [Neutral Cellulase]: 1%, pH = 7, Time, 30 min., Temperature, 50 °C, M: L, 1:10. Sol–gel Treatment Conditions: Curing Temperature, 150˚C; Curing Time, 15 min. * Washed one cycle according to AATCC Test Method (61-1989).

**Table 4:** Effect of activation of PET/C blended fabrics on its antimicrobial activity.

Reagents	Antimicrobial Assessment		
	*Staphylococcus aureus*	*Escherichia coli*	*Candida albicans*
PET/C (Parent)	−ve	−ve	−ve
PET/C → N-Cell (Control)	−ve	−ve	−ve
PET/C → A-Cell (Control)	−ve	−ve	−ve
PET/C → N-Cell → ZnO NPs	71.5	71.2	82.9
PET/C → A-Cell → ZnO NPs	70.8	71.9	65.4

Enzymatic Treatment Conditions: [Acid Cellulase]: 1%, [Acetic Acid], 1.0 g/l, pH = 4.5, Time, 30 min., Temperature, 50 °C, M: L, 1:10. [Neutral Cellulase]: 1%, pH = 7, Time, 30 min., Temperature, 50 °C, M: L, 1:10. Sol–gel Treatment Conditions: Curing Temperature, 150˚C; Curing Time, 15 min. * Washed one cycle according to AATCC Test Method (61-1989).

### Ultraviolet protection properties

The effect of activation of cotton and PET/C blend fabrics either with acid or neutral cellulases before loading with sol ZnO NPs, on UV protection efficiency was investigated. The rate of UV protection was measured and expressed via UPF values that are listed in Tables 5 and 6. It was found that the UPF factors for parent cotton, PET/C blend fabrics are poor and equal to 11.7 and 13.6 respectively. Activation with cellulases followed by the ZnO NPs deposition onto the above mentioned fabrics led to a significant increase in UPF factor to the level corresponding to UPF rating of 50+, which assigns the excellent UV protection, After five washing cycles, these results imply provide 50+ laundering durability of fabrics activated with neutral and acid enzymes and loaded with ZnO NPs. It was found that, cotton and PET/C blended fabrics activated with acid enzymes and loaded with ZnO NPs showed better UV protection efficiency compared to neutral enzymatic treated ones 50+, which assigns the excellent UV protection. The UV protection efficiency of these fabrics is higher even after five washing cycles, indicating the excellent laundering durability.

**Table 5:** Effect of activation of cotton fabrics on its UPF values.

Fabric	UPF Assessment at Number of washing cycles:			
	1**		5**	
	Value	Rating	Value	Rating
Cotton (Parent)	11.7	Poor	11.7	Poor
Cotton → Neutral Cellulase (Control)	10.6	Poor	10.6	Poor
Cotton → Acid Cellulase (Control)	10.2	Poor	10.2	Poor
Cotton → Neutral Cellulase → ZnO NPs	69.3	Excellent	62.4	Excellent
Cotton → Acid Cellulase → ZnO NPs	63.3	Excellent	55.7	Excellent

Enzymatic Treatment Conditions: [Acid Cellulase]: 1%, [Acetic Acid], 1.0 g/l, pH = 4.5, Time, 30 min., Temperature, 50 °C, M: L, 1:10. [Neutral Cellulase]: 1%, pH = 7, Time, 30 min., Temperature, 50 °C, M: L, 1:10. Sol–gel Treatment Conditions: Curing Temperature, 150˚C; Curing Time, 15 min. * Washed one and five cycles according to AATCC Test Method (61-1989).

**According to Australia (AS) / New Zealand (NAS) Standard No. 4399 (1996).

**Table 6:** Effect of activation of PET/C blend fabrics on its UPF values.

Fabric	UPF assessment at number of washing cycles:			
	1**		5**	
	Value	Rating	Value	Rating
PET/C (Parent)	13.6	poor	13.6	Good
PET/C → Neutral Cellulase (Control)	17.8	Good	17.8	Good
PET/C → Acid Cellulase (Control)	15.3	Good	15.3	Good
PET/C → Neutral Cellulase → ZnO NPs	73.5	Excellent	68.5	Excellent
PET/C → Acid Cellulase → ZnO NPs	91.1	Excellent	86.7	Excellent

Enzymatic Treatment Conditions: [Acid Cellulase]: 1%, [Acetic Acid], 1.0 g/l, pH = 4.5, Time, 30 min., Temperature, 50 °C, M: L, 1:10. [Neutral Cellulase]: 1%, pH = 7, Time, 30 min., Temperature, 50 °C, M: L, 1:10. Sol–gel Treatment Conditions: Curing Temperature, 150˚C; Curing Time, 15 min. * Washed one and 5 cycles according to AATCC Test Method (61-1989). **According to Australia (AS)/New Zealand (NAS) Standard No. 4399 (1996).

## Conclusion

The current study describes an easy and Eco sustainable approach for increasing the binding efficiency of sol ZnO NPs to cotton and PET/C blended fabrics. This eco-technique involves enzymatically treating the fabrics with acid and neutral cellulases before loading them with ZnO NPs generated using the sol–gel process. These finished fabrics were evaluated using SEM, EDX, and FT-IR spectroscopy, demonstrating that ZnO NPs are chemically attached to fabrics. The effect of surface activation prior to loading sol ZnO NPs on antibacterial efficacy and UV protection efficiency of fabrics was investigated. Cotton and PET/C fabrics activated with acid cellulase before loading with ZnO NPs shown superior antibacterial and UV protection characteristics as compared to neutral cellulase treated fabrics. Even after five washing cycles, activated fabrics had exceptional antibacterial activity and UV protection efficacy, indicating great laundry durability. In general, the proposed technique in this study confirmed the feasibility of using biological surface activation technology as an eco-friendly method to improve the chemical attachment of sol ZnO NPs to cotton and PET/C blended fabrics. The new study’s combination of nano and bio technologies provides textile finishers with an economical and environmentally friendly alternative to traditional chemical and mechanical finishing processes employed in the textile industry.

## Data Availability

The authors declare that, the data supporting the findings of this study are available within the paper. Should any raw data files be needed in another format they are available from the corresponding author upon request.
